# Incidence Rate and Risk Factors for Tuberculosis among People Living with HIV: A 2015–2017 Cohort from Tashkent, Uzbekistan

**DOI:** 10.3390/ijerph18115746

**Published:** 2021-05-27

**Authors:** Dilbar Sadirova, Ruzanna Grigoryan, Nargiza Parpieva, Venera Barotova, Aleksandr Trubnikov, Lola Kalandarova, Jamshid Gadoev, Davron Mukhtarov, Mariana Buziashvili, Nestani Tukvadze, Arax Hovhannesyan, Andrei Dadu

**Affiliations:** 1 Center of Phthisiology and Pulmonology of Tashkent City, Tashkent 100043, Uzbekistan; aleksandr.trubnikov.dots@mail.ru (A.T.); lola_nur69@mail.ru (L.K.); 2 TB Research and Prevention Center, Yerevan 0023, Armenia; ruzanna.grigory@gmail.com; 3 Republican Specialized Scientific Practical Medical Center of Phthisiology and Pulmonology, Tashkent 100043, Uzbekistan; nargizaparpieva@gmail.com; 4 Tashkent City AIDS Center, Tashkent 100043, Uzbekistan; vinera.barotova@minzdrav.uz; 5 World Health Organization (WHO) Country Office in Uzbekistan, 16 Tarobiy Street, Tashkent 100100, Uzbekistan; gadoevj@who.int; 6 Tashkent Institute of Postgraduate Medical Education, Tashkent 100043, Uzbekistan; al-sarvar@mail.ru; 7 National Center for Tuberculosis and Lung Diseases, Tbilisi 01790101, Georgia; buziashvili.mari@gmail.com (M.B.); marikushane@yahoo.com (N.T.); 8 World Health Organization, Regional Office for Europe, UN City, Marmorvej 51, DK-2100 Copenhagen, Denmark; hovhannesyana@who.int (A.H.); dadua@who.int (A.D.)

**Keywords:** tuberculosis, HIV, Uzbekistan, Central Asia, incidence rates, operational research, SORT IT

## Abstract

People living with the human immunodeficiency virus (PLHIV) have a higher risk of developing active tuberculosis (TB) disease, and TB remains a major cause of death in PLHIV. Uzbekistan is facing a substantial TB epidemic, which increases the risk of PLHIV developing active TB. Our retrospective cohort study aimed to evaluate the incidence rate and assess the risk factors for developing active TB among PLHIV. We collected secondary data extracted from medical charts of all patients, newly diagnosed at the AIDS Center in Tashkent, during the period of 2015–2017. The incidence rate of TB among PLHIV was 5.1 (95% CI: 4.5–6.0) per 1000 person/month. Adjusted regression analysis showed three major risk factors for TB, namely, being less than 15 years old (hazard ratio (HR) 5.83; 95% CI: 3.24–10.50, *p* value = 0.001),low CD4 count (adjusted hazard ratio(aHR) 21.0; 95% CI: 9.25–47.7, *p* value < 0.001), and antiretroviral therapy (ART) interruption/not receiving ART (aHR 5.57; 95% CI: 3.46–8.97 and aHR 6.2; 95% CI: 3.75–10.24, *p* value < 0.001, respectively) were significantly associated with developing active TB among PLHIV. Our findings indicate that taking prescribed ART without interruptions and maintaining CD4cell counts higher than 320 cells/μL are essential to prevent the development of active TB among PLHIV.

## 1. Introduction

Human immunodeficiency virus/acquired immunodeficiency syndrome (HIV/AIDS) is the second most deadly infectious disease after tuberculosis (TB) globally. At the end of 2019, there were 38.0 million people living with HIV (PLHIV) worldwide, out of whom 1.7 million were newly infected and 690,000 died from HIV-related diseases in the same year [1]. TB remains a leading cause of death in PLHIV, even among patients receiving antiretroviral therapy (ART). In 2018, an estimated 9% of the incident TB cases was reported among PLHIV. In addition, 300,000 patients with HIV died from TB in the same year [2,3]. The risk of developing TB in PLHIV is 20 to 30 times higher compared to the risk amongst the general population [2]. The lifetime risk of TB in the general population is between 10% to 15%, whereas PLHIV have a5–10% annual risk of developing TB [4]. To reduce the risk of developing TB among PLHIV, the World Health Organization (WHO) recommends TB preventive treatment along with ART for both adults and adolescents living with HIV [5,6].

Despite international and domestic efforts to mitigate the impact of HIV epidemics, HIV is still growing in the Eastern Europe and Central Asia region with an annual 30% increase in HIV infections during the periodoffrom2010 to 2018. This region is also facing a substantial TB epidemic, with a growing burden of serious TB drug resistance [2]. The Republic of Uzbekistan, a country in Central Asia, has an estimated HIV prevalence of 0.15% (52,000 cases) among the general population. In 2018, the Joint United Nations Programme on HIV/AIDS (UNAIDS) estimated that there were 16 incident cases of HIV per 100,000 adult population in the country. In the same year, around half (51%) of PLHIV received ART, and the estimated prevalence of active TB among PLHIV was 22% (1200) [7].

Uzbekistan administers isoniazid preventive therapy (IPT) along with ART to PLHIV according to national guidelines, which are in line with WHO [5,6,8]. The incidence rate and risk factors for developing TB among PLHIV have not previously been explored in Uzbekistan. We therefore aimed to evaluate the incidence rate and assess the risk factors for developing active TB among PLHIV who received clinical care at the main referral AIDS Center in the capital city, Tashkent.

## 2. Materials and Methods

### 2.1. Study Design

We conducted a retrospective cohort study using secondary data of all patients who were referred to the AIDS Center in Tashkent City between 2015 and 2017 and were newly diagnosed and enrolled in HIV care.

### 2.2. Study Setting—General, Study Site and Study Period

Uzbekistan is a lower-middle income country located in Central Asia with a population of 32 million. It includes twelve provinces (oblasts), the autonomous Republic of Karakalpakstan and the capital city of Tashkent.

### 2.3. HIV Care

The Ministry of Health developed the National HIV Program in collaboration with national and international partners. The Republican AIDS Center is responsible for coordinating, monitoring and evaluating HIV care throughout the country. HIV care is provided at all levels of the health care system, including polyclinics (infectious disease cabinets); city AIDS centers, such as the Tashkent City AIDS Center; regional AIDS centers; and the Republic AIDS Center. HIV care is provided in accordance with the National HIV treatment and diagnostic protocol, which was recently updated based on the WHO HIV treatment guidelines [9]. The national protocol covers the provision of IPT and the management of HIV/TB coinfection. The cooperation between HIV and TB programs is regulated by Order No. 277 of the Ministry of Health.

All HIV-positive patients undergo standard TB screening and diagnostic procedures. TB screenings with chest X-rays are provided every year as part of routine HIV management. All identified presumptive TB cases undergo bacteriological examinations with sputum smear microscopy and Xpert MTB/Rif. All bacteriologically or clinically confirmed cases are sent to a TB healthcare facility for TB treatment. If active TB is ruled out, an HIV-infected person receives IPT for six months every three years. Those who are found to have had a previous TB treatment history or comorbidities that are contraindications for IPT, as well as those with individual intolerance to isoniazid or who refuse to get IPT, are excluded from TB preventive treatment. The IPT and ART are both administered in the same health care facility.

### 2.4. TB Care

The National Tuberculosis Programme delivers free TB services in all territories of Uzbekistan through the Republican Specialized Scientific-Practical Medical Center of Phthisiology and Pulmonology (RSSPMCPP) located in Tashkent city. At the provincial TB healthcare facilities, TB services are provided under the supervision of the Ministry of Health and province state health departments. Uzbekistan has been practicing the WHO-recommended Directly Observed Treatment Strategy (DOTS) since 1998, and it reached countrywide coverage in 2005. The intensive phase of Directly Observed Treatment (DOT) is administered in the secondary-level TB facilities, while the continuation phase is provided in the primary health care facilities. The country is covered by a network of TB laboratories, including two National Reference, five bacteriological and more than 200 smear microscopy laboratories. TB care for both drug-susceptible and drug-resistant TB is provided according to ministerial orders in line with WHO-recommended guidelines [10,11].

### 2.5. Study Population

The study included people who were diagnosed with HIV from 2015 to 2017 in the Tashkent City AIDS Center. The exclusion criterion was active TB disease at the moment of HIV diagnosis or revealed within three months from HIV diagnosis. Information on HIV status and clinical and demographic data were collected at the AIDS Center, while the patients’ history on active TB diagnosis was followed in patient charts at the Republican Specialized Scientific-Practical Medical Centre of Phthisiology and Pulmonology (RSSPMCPP), where all presumptive TB cases were referred for TB diagnosis and treatment. Histories on TB status were re-evaluated in November 2020, giving a minimum 36- and maximum 54-month follow-up time for the first and last enrolled PLHIV.

### 2.6. Sources of Data

All data were extracted from the hospital files and patient records of the Tashkent City AIDS Center and Republican Specialized Scientific-Practical Medical Centre of Phthisiology and Pulmonology.

### 2.7. Data Collection and Validation

All the required data extracted from patient medical records and electronic registers were entered into standard electronic records developed using EpiData application (version 3.1 EpiData Association, Odense, Denmark). Inconsistencies were resolved by analyzing the source documents.

### 2.8. Data Variables

The study outcome of interest was the time to diagnosis of active TB disease, defined as a patient in whom TB was confirmed by bacteriology or diagnosed by a clinician. Explanatory variables included age, sex, TB history, current TB diagnosis, history of incarceration, drug use, alcohol use, smoking, concomitant diseases, CD4 cell count at the time of HIV diagnosis and ART uptake.

### 2.9. Analysis and Statistics

A descriptive analysis was used to examine patient demographic and clinical characteristics, and the results are presented as percentages and numbers. The age variable was converted into three categories split at the age of 14 and 40 years. CD4 cell count at the time of HIV diagnosis was dichotomized using the median value. Follow-up time was defined as the period from the 3rd month since the date of HIV diagnosis until the date of TB diagnosis, death or end of study period (30 December 2019) (right censored). All the variables were examined for their association with the outcome of interest (to evaluate their potential as a predictor) by computing the hazard ratio (HR), confidence interval (CI) for deviation from 1 and *p* value using univariate Cox regression analysis. We examined the time to TB diagnosis by constructing Nelson–Aalen (cumulative hazards) survival curves by categories for each of the explanatory variables. To assess the joint effect of variables associated with time to TB diagnosis, variables associated with each of the outcomes in the univariate Cox proportional hazard model at *p* < 0.1 level were included in a multivariate model using a forward stepwise procedure based on ascending *p* value in the univariate analysis. Age and gender were treated as a prior variables for inclusion. A model fit was assessed using the likelihood ratio test (LRT). Interactions based on plausible relationships of covariates associated with the outcome of interest were assessed in the final model. Adjusted hazard ratios and their 95% CI were reported from the final model. The validity of the assumption of proportional hazard was assessed using Schoenfeld’s global test. All statistical tests were two-tailed. Statistical analysis was completed using Intercooled Stata software version 15 (Stata Corp., College Station, TX, USA).

## 3. Results

A total of 1101 new HIV cases were registered in the City AIDS Center of Tashkent, between January 2015 and December 2017, out of whom 79 had active TB at the time of HIV diagnosis and another 12 patients were diagnosed with TB within the 3 months after HIV diagnosis. All those patients were excluded from the study population.

Within the cohort of 1010 PLHIV evaluated for the study, the mean age was 37.9 years (SD 11.0) and 579 (57.3%) were male, 26 (2.7%) had a previous history of TB and 96 (9.7%) had a history of imprisonment. Drug, alcohol and tobacco use accounted for 52 (5.3%), 169 (17.1%) and 282 (28.6%) cases, respectively. The majority of patients, 825 (81.8%), had one or more types of concomitant disease. Of these, the type of comorbidity was known for 788 patients and the most frequent comorbidities were TORCH syndrome 147 (18.65%), hepatitis C 139 (17.64%), dermatitis 129 (16.37%) and peptic ulcer 83 (10.53%). A high proportion of TORCH syndrome, 139 (94, 56%), was seen among PLHIV over 14 years of age.

Among 1010 PLHIV enrolled in the study, 176 (17.4%) developed active TB over the 33,900 person/month (p/m) follow-up period, with a TB incidence rate of 5.1 (95% CI: 4.5–6.0) per 1000 p/m.

TB incidence rates were found to be higher among males at 6.6 per 1000 p/m compared to females 3.48 per 1000 p/m. Among those with a CD4 cell count of less than 320 cell/μL, the TB incidence rate was 11.3 per 1000 p/m (*n* = 170, 33.5.0%); among those with any type of comorbidity, the TB incidence rate was 6.75 per 1000 p/m (*n* = 173, 21.0%); and among those who interrupted or did not receive prescribed ART, the TB incidence rate was 10.72 and 9.06 per 1000 p/m, respectively (*n* = 94, 36.0% and *n* = 54, 30.7%). Table 1 show factors associated with developing TB among PLHIV during the follow-up period.

In an unadjusted analysis, the risk factors of developing TB in PLHIV were as follows: being less than 15 years old (HR 5.83; 95% CI: 3.24–10.50, *p* value = 0.001), a history of incarceration (HR 2.09; 95% CI: 1.41–3.10, *p* value < 0.001), drug use (HR 1.97; 95% CI: 1.27–3.37, *p* value = 0.003), alcohol consumption (HR 2.69; 95% CI: 1.16–3.34, *p* value <0.013), any comorbidities (HR 14.00, 95% CI: 4.47–43.85, *p* value < 0.001), low CD4 cell count (HR 34.28; 95% CI: 14.74–79.1, *p* value < 0.001) and history of interruption (HR 10.72; 95% CI: 6.54–17.55, *p* value < 0.001) or refusal (HR 9.06; 95% CI: 5.37–15.29, *p* value < 0.001) of ART therapy.

The final adjusted regression analysis showed three major risk factors that remained significant for developing TB: being less than 15 years old (adjusted hazard ratio (aHR) 7.88; 95% CI: 4.30–14.39, *p* value = 0.001), low CD4 count (aHR 23.3; 95% CI: 10.26–52.73, *p* value < 0.001) and ART interruption/no-ART (aHR 7.00; 95% CI: 4.26–11.49 and aHR 7.60; 95% CI: 4.48–12.88, *p* value < 0.001, respectively).

**Figure 1:**
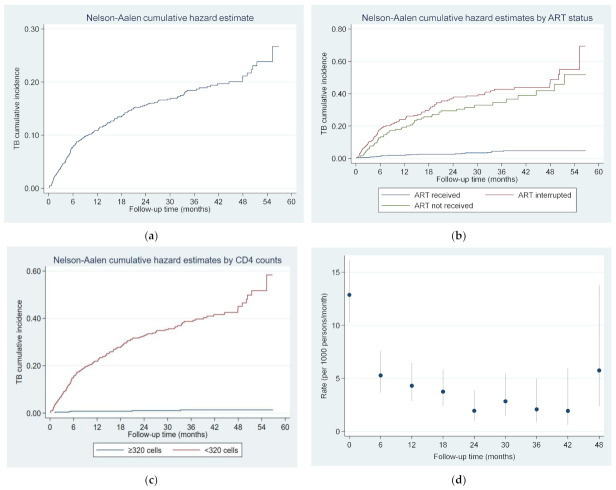
(**a**) Nelson–Alen cumulative hazard estimates for developing TB among people newly diagnosed with HIV from 2015 to 2017 in Tashkent, Uzbekistan. (**b**) Nelson–Alen cumulative hazard estimates for developing TB among people newly diagnosed with HIV from 2015 to 2017, by ART status. (**c**). Nelson–Alen cumulative hazard estimates for developing TB among people newly diagnosed with HIV from 2015 to 2017 in Tashkent, Uzbekistan, by CD4 cell count. (**d**) Six-monthly average TB incidence rates among 1010 people newly diagnosed with HIV, over a 5-year follow-up period; vertical lines indicate 95% confidence interval. Incidence rate was estimated for 6-month time intervals; for example, the rate at month 6 indicates the period greater than 6 months and less than or equal to 12 months.

Figure 1a shows cumulative hazard estimates for developing TB among all PLHIV, while Figure 1b,c show cumulative hazard estimates desegregated by ART status and CD4 count at the time of TB diagnosis. Cumulative incidence rates of tuberculosis among 1010 PLHIV were highest in first 6 months (5.2 per 1000p/m), remaining comparatively high from the 12th month to the 18th month (Figure 1d).

## 4. Discussion

Our study was the first in Uzbekistan to describe the TB incidence rate among PLHIV and assess factors associated with increased incidence. The TB incidence rate found in our study (5.1 per 1000 p/m) was not unexpected taking into account general factors, such as Uzbekistan being a high TB burden and medium socio-economic (by World Bank classification) level country [12], and various individual level factors as described below.

In our study, we found higher TB incidence among males compared to females. In general, males show an increased TB prevalence compared with females [13,14], and some studies have also shown that male sex is associated with a higher risk of TB incidence among PLHIV, independent of any ART regimen [15]. Even though in our study the adjusted analysis did not show a major association between male sex and higher TB incidence, there was a strong association between male sex and lower CD4 cell count at diagnosis. These sex differences can be explained by different cultural and/or economic factors. For example, men are less likely to make time to receive medical care compared with women [16].

The TB incidence rate among PLHIV who interrupted or did not receive prescribed ART was ten times higher compared to those who were on ART. Cumulative hazard estimates for TB remained very low in PLHIV on ART in comparison to the interrupted/non-ART groups, with interruption showing a slightly higher risk for TB compared to those not receiving ART at all. Our findings are consistent with previous studies showing that high CD4 cell counts are protective against the development of active TB [17]. By destroying cell-mediated immunity, HIV increases the risk of TB either through new infection with *Mycobacterium tuberculosis* or through reactivation of latent TB infection. ART is a protective factor as it restores immunity, with some studies showing that this is not just due to the medication, but rather due to its effect on restoring CD4 cell counts [18].

One additional factor that remained significant in the adjusted analyses was being less than 15 years old, which is very much consistent with recent systematic reviews and meta-analyses. In addition, the literature also shows that the risk for TB remains elevated among children compared to the elderly population, even with excellent ART coverage [19,20].

Comorbidities were not found to be significant risk factor in adjusted analysis. An unexpectedly high proportion of TORCH syndrome was seen among the non-pediatric population, which needs to be further evaluated.

We found that the cumulative incidence of active TB in our study population was highest in the first year. In PLHIV with advanced disease, tuberculosis can manifest itself in a non-specific or atypical way, and this can make the initial diagnosis difficult. In addition, clinical manifestations of TB can become obvious soon after initiating ART because of the immune reconstitution inflammatory syndrome [21]. Due to these challenges, it may take some time to diagnose TB after patients are enrolled for HIV care, and this can explain the higher incidence of TB usually seen in first months after HIV diagnosis [22]. Although all active TB cases revealed within the three months after HIV diagnosis were excluded from the study, the TB incidence rate still remained higher within the first months. It may also be that the CD4cell count is still low at this time, causing the patients to remain at high risk of TB.

In our retrospective study, information on several important potential risk factors was missing in the charts. Some studies have shown that low hemoglobin levels [23], increased HIV viral load [24], genetic factors [25] and WHO-defined HIV clinical stages [26] are important risk factors for incidents of TB. We also could not extract detailed socio-demographic data to examine important and vulnerable sub-populations, but other studies have shown that being a prisoner [27], a migrant [28], a health care worker [29] and a household or close contact of an active index TB case [30] can substantially increase the risk of incident TB among PLHIV.

One of the major limitations of our study was that we could not collect information on IPT consumption. Uzbekistan has introduced IPT, and the reported coverage of 6 months of IPT among PLHIV was 60% and 64% in 2016 and 2017, respectively. Unfortunately, the patient charts reviewed in our study did not include details on whether patients had received IPT or not. While prescription IPT is documented, while DOT or any means of IPT receipt is not. The cumulative incidence of TB among PLHIV in our study might have been influenced by IPT consumption, but we could not evaluate its individual potential protective effect.

## 5. Conclusions

Having a high CD4 count and using ART were protective against incident TB among PLHIV in our study population in Uzbekistan. More effort is needed to ensure patients are put on ART as soon as possible and that there is close monitoring of CD4cell counts. Study results will be made available to both TB and HIV programs as well as the Ministry of Health of Uzbekistan to improve domestic efforts for controlling HIV/TB coinfection. We also recommend further analysis of the TB diagnostic cascade among PLHIV to learn how effective the current system is in identifying HIV and TB cases, what the potential factors are that cause low ART coverage, what the IPT compliance rate is and its potential role in preventing TB in this vulnerable and at-risk population.

## Figures and Tables

**Table 1:** Factors associated with TB development among people newly diagnosed with HIV from 2015 to 2017, Tashkent, Uzbekistan.

Variable	Total			Developed TB		TB Rate (1000p/m)	Unadjusted Analysis			Adjusted Analysis		
	*N*		%	*N*	%		HR	95% CI	*p* Value	aHR	95% CI	LRT *p* Value
Age in years												
<15 years	20		2.0	13	65.0	26.9	5.83	(3.24–10.50)	<0.001	7.88	(4.30–14.39)	<0.001
15–39 years	584		57.8	85	14.6	4.14	1			1		
≥40 years	406		40.2	78	19.2	6.05	1.4	(1.03–1.91)	0.032	1.12	(0.81–1.54)	
Sex												
Male	579		57.3	123	21.2	6.6	1			1		
Female	431		42.7	53	12.3	3.48	0.54	(0.39–0.75)	<0.001	0.87	(0.62–1.21)	0.399
History of TB treatment												
Yes	26		2.7	4	15.4	4.38	1					
No	940		97.3	171	18.2	5.45	1.21	(0.45–3.25)	0.623			
Missing	44											
History of being in prison												
Yes	96		9.7	30	31.3	10.28	2.09	(1.41–3.10)	<0.001			
No	892		90.3	143	16.0	4.72	1					
Missing	22											
CD4 count in numbers												
>320 cells/μL	497		49.5	6	1.2	0.32	1			1		
<320 cells/μL	508		50.5	170	33.5	11.3	34.28	(14.74–79.13)	<0.001	23.3	(10.26–52.73)	<0.001
Missing	5											
Concomitant diseases												
Yes	825		81.8	173	21.0	6.75	14	(4.47–43.85)	<0.001			
No	183		18.2	3	1.6	0.44	1					
Missing	2											
Drug use												
Yes	52		5.3	15	28.8	9.21	1.97	(1.27–3.37)	0.003			
No	937		94.7	148	15.8	4.66	1					
Alcohol use												
Yes	169		17.1	56	33.1	11.17	2.69	(1.16–3.34)	0.013			
No	819		82.9	106	12.9	3.74	1					
Smoking												
Yes	282		28.6	61	21.6	4.18	1.55	(1.13–2.13)	0.007			
No	704		71.4	101	14.3	6.63	1					
ART												
Received		464	51.5	19	4.1	1.1	1					
Interrupted		261	29.0	94	36.0	12.37	10.72	(6.54–17.55)	<0.001	7.00	(4.26–11.49)	<0.001
Not received		176	19.5	54	30.7	10.94	9.06	(5.37–15.29)	<0.001	7.60	(4.48–12.88)	
Missing		109										

HR = hazard ratio, aHR = adjusted hazard ratio, p/m = person/month, CI = confidence interval, LRT = likelihood ratio test, ART = anti-retroviral therapy.

## Data Availability

The data that support the findings of this study are available from the corresponding author, upon reasonable request.

## Open Access Statement

In accordance with WHO’s open-access publication policy for all work funded by WHO or authored/co-authored by WHO staff members, the WHO retains the copyright of this publication through a Creative Commons Attribution IGO licence (http://creativecommons.org/licenses/by/3.0/igo/legalcode (accessed on 12 February 2021)) which permits unrestricted use, distribution and reproduction in any medium provided the original work is properly cited.
